# Researchers’ experiences with patient engagement in health research: a scoping review and thematic synthesis

**DOI:** 10.1186/s40900-023-00431-8

**Published:** 2023-04-10

**Authors:** Marie-Mychèle Pratte, Sophie Audette-Chapdelaine, Anne-Marie Auger, Catherine Wilhelmy, Magaly Brodeur

**Affiliations:** 1 Faculté de médecine et des sciences de la santé, Université de Sherbrooke, Sherbrooke, QC Canada; 2 Département de médecine familiale et de médecine d’urgence, Université de Sherbrooke, Sherbrooke, QC Canada; 3 Comité stratégique patient-partenaire, Centre de Recherche du CHUS, Université de Sherbrooke, Sherbrooke, QC Canada; 4 Centre de recherche du CHUS, Université de Sherbrooke, Sherbrooke, QC Canada

**Keywords:** Patient and public involvement, Researcher, Patient engagement, Experience, Qualitative research, Scoping review, Thematic synthesis, Health

## Abstract

**Context:**

Implicating patients in research is gaining popularity around the world and is now the reference of many funding agencies. Understanding these partnerships is necessary to grasp this new reality. The experiences of researchers who have involved patient-partners (PPs) in health research are important for a better understanding of these practices.

**Objective:**

This study aimed to identify and analyze the existing qualitative scientific literature on the experiences of academic researchers involved in health research with patient engagement (PE).

**Design:**

A scoping review of the available literature with an inductive thematic synthesis, guided by the methodological framework of Arksey and O’Malley.

**Data collection:**

A search strategy was developed to include keywords relating to researchers, patient-partners, experiences, and the qualitative methodologies of the targeted studies. Five databases were searched using the EBSCO-host engine. The search results were screened by four reviewers to only include articles written in English on the topic of the experience of academic researchers having worked with PPs in health research based on qualitative studies or mixed-methods studies with a distinct qualitative section.

**Analysis:**

Articles included were charted for general information. All “results” sections were coded line by line. These codes were organized inductively to form descriptive and analytical themes. This led to the synthesis of the ideas found in the selected articles.

**Results:**

The search strategy yielded 7616 results, of which 2468 duplicates were removed. The remaining 5148 articles were screened, resulting in the exclusion of 5114 off-topic studies. The remaining 29 full-text articles were evaluated for inclusion from which 5 additional studies were identified. The final selection consisted of 11 articles that met all the criteria. These articles were published between 2009 and 2019. Five general themes inductively emerged from the analysis: the understanding of PE, motivations, contexts, attitudes, and practical aspects of PE that are central to researchers.

**Conclusion:**

This scoping review provides a better understanding of the experiences of researchers who have implemented patient partnerships in health research projects. Our findings reveal many positive elements central to health researchers’ discourses about PE, but they provide insights into the challenges and postures of resistance. This knowledge can support the development of empirically sound improvements in PE practices.

**Supplementary Information:**

The online version contains supplementary material available at 10.1186/s40900-023-00431-8.

## Abstract (summary)

The practice of involving patients in research is gaining popularity around the world and is now the reference for many institutions and funding agencies. The individual experiences of researchers who have involved patient-partners in health research are essential for a better understanding of how these relationships unfold within research teams, and allows for identifying the challenges and best practices. This review’s objective is to identify and analyze the existing scientific literature focused on the experiences of academic researchers involved in health research with patient engagement (PE). A close screen of 7616 search results yielded 11 scientific peer-reviewed articles corresponding to all inclusion criteria for further analysis. Five general themes emerged from the analysis: (1) the understanding of PE, (2) the motivations for PE, (3) the contexts of PE, (4) the attitudes toward PE, and (5) the practical aspects of PE that are central to health researchers. This analysis provides a novel summary of the current knowledge on the in-depth experiences of researchers who have implemented patient-partnerships in health research projects. Beyond many positive elements central to health researchers, our analysis also revealed the challenges and postures of resistance. Both positive and negative perspectives on PE help to draw a better understanding of these collaborations. This knowledge can support the development of empirically sound improvements in PE.

**Supplementary Information:**

The online version contains supplementary material available at 10.1186/s40900-023-00431-8.

## Background

Existing literature has well established the positive benefits and impacts associated with patient participation in research [1, 2]. For example, patient participation has lead to developing projects and innovations focused on patient needs [1, 3–5]. It has also facilitated the implementation of innovations and the acceptability of scientific data [3, 6]. In health research, where patient participation has become the gold standard, it has been associated with an increased quality of care and research [7–10]. Renowned international leaders on this topic include INVOLVE a program by the National Institute for Health Research in the United Kingdom (UK) and the Patient‐Centered Outcomes Research Institute (PCORI) in the United States. PCORI-affiliated authors, Forsythe et al. [3], concluded that patient engagement (PE) is associated with improved acceptability, feasibility, rigor, and better data quality, as well as projects that are globally more relevant. The literature suggests that efforts should be invested in understanding how partnerships with patients can produce positive impacts in health research and how to facilitate the engagement of patient-partners (PP) in health research [9, 11]. Although we know a lot about the experience of PPs in health research [12], our understanding of the experience of researchers collaborating with PPs in health research is limited.

PE in health research occurs when PPs are involved in research co-production [5, 6, 13]. In this context, the word “patient” is understood as any person having experienced health issues, either personally or as a caregiver [5]. PE is meant to build to partnerships between patients and academic researchers with the objective of constructing a “meaningful and active collaboration in governance, priority setting, conducting research and knowledge translation” [5], rather than participating in health research projects only as research subjects [5]. Different terminology is used internationally to refer to what this review calls PE, such as the term “patient and public involvement” (PPI) or “expert by experience”, which are common in the United Kingdom. As this scoping review is based in a Canadian university, the expression “patient engagement” (PE) is used and is considered a synonym for the same expression.

This scoping review aims to produce a qualitative summary of the current knowledge on the experiences of researchers who involve PPs in health research projects.

## Methods

### Study design

A scoping review of the peer-reviewed published literature with an inductive thematic synthesis was performed, guided by the methodological framework of Arksey and O’Malley [14], and enhanced by the perspectives of Levac et al. [15] and Peters et al. [16]. The Enhancing transparency in reporting the synthesis of qualitative research (Additional file 1: ENTREQ) statement checklist by the Enhancing the Quality and transparency Of health Research (EQUATOR) network was also used to safeguard the quality of our reporting.

### Research question

The research question guiding this review is: “What are the experiences of researchers with PE in health-related research?”.

### Eligibility criteria

Studies were screened for eligibility according to the following inclusion criteria: study participants who are academic researchers experienced with PE in health research projects, articles in which one of the main objectives was to study the experience of the researchers, articles that used qualitative methods, and articles written in English. To increase useful data, mixed-method studies were also included if a qualitative section could be independently analyzed. No limits were applied regarding the year of publication of the articles. The studies eligibility was lastly screen for the following exclusion criteria: articles not reporting first level data and articles that were not peer reviewed.

### Search strategy

The search strategy was developed by MMP in collaboration with an academic health librarian and the ongoing teamwork, validation, and discussions with SAC, AMA, and MB. We also integrated many of the keywords suggested by Rogers et al. [17] and added those found during preliminary database tests. The key concepts grounding the search strategy were *researcher, experience, patient-partner,* and *qualitative research.* Our search strategy was developed with free-form keywords and controlled keywords (MeSH) adapted for each database.

**Fig. 1:**
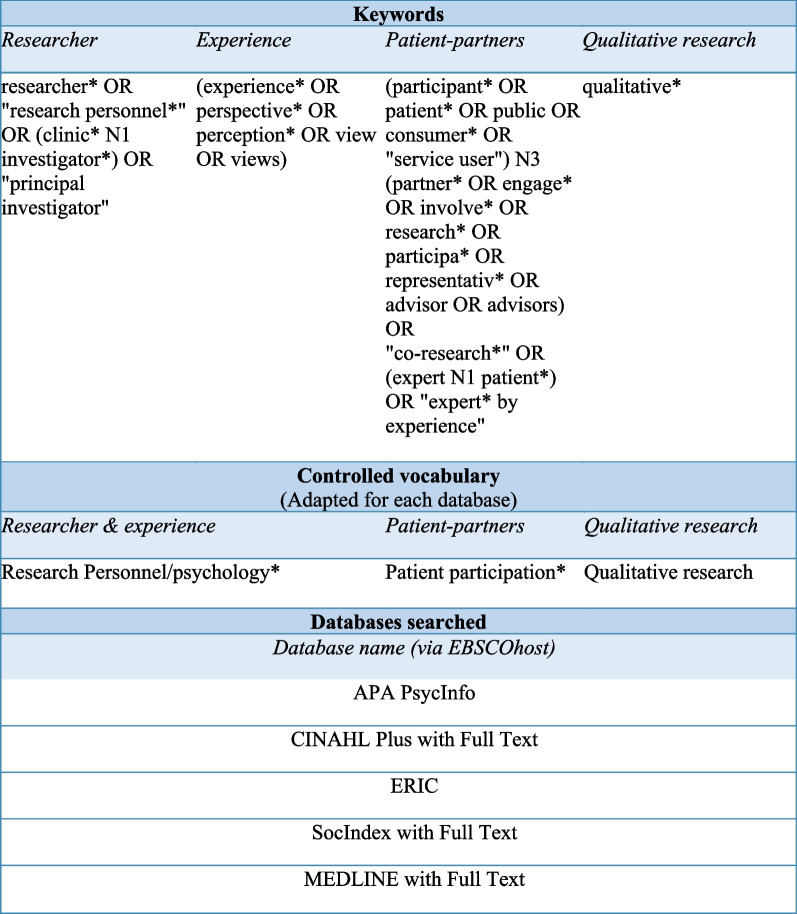
Summary of the search strategy

On August 18, 2022, the following five databases were searched by MMP via the EBSCOhost search engine: American Psychological Association (APA) PsycInfo, Cumulative Index to Nursing and Allied Health Literature (CINAHL) Plus with Full Text, Education Resources Information Center (ERIC), Medical Literature Analysis and Retrieval System Online (MEDLINE) with Full Text, and SocIndex with Full Text. Google Scholar was also searched on August 19, 2022, limited to the first 10 pages of results. The search strategy is presented in Fig. 1.

### Study selection

All found articles were extracted and imported into *ReadCube Papers* reference management software (v4.34.2142). First, duplicates were removed. Second, the titles and abstracts were screened to determine eligibility for a full-text review by MMP. Full-text screening of all remaining articles was completed by MMP and cross-validated by SAC, MB, and AMA. Following the same process, the references cited in each included article were scanned for other studies that could be included in the review. The final selection was agreed upon by consensus of all team members after discussion on the eligibility of specific articles, when relevant. A quality assessment of the 11 selected articles was not conducted since there are no appropriate standardized criteria for assessing qualitative research with a range of designs [18] and since quality assessment is often described as outside of the framework of scoping reviews [14, 15].

### Data extraction and analysis

A charting form was used to assist team discussion and approbation as a support to the full-text screening and for the descriptive analysis. For each article, the information extracted in a charting form, inspired by the charting forms developed by Joanna Briggs Institute [19], Lauzon-Schnittka et al. [12] and Arnstein et al. [20], were: authors, year of publication, country of origin, title, aim, type of evidence source, participant number and demographics, whether a specific population was targeted, definitions of PPs and PE, whether PPs were engaged in the study, whether PPs were coauthors or otherwise acknowledged, interview guide subjects, and main findings. If the data items were not specified, the field was left blank. The extraction was completed by MMP and validated by team members.

The Results/Findings section of each article was coded line by line in Microsoft Word (v.16.70) [21]. The other sections of the articles provided a better understanding of the studies but were not used for the line-by-line analysis. The line-by-line coding and theme identification followed the thematic synthesis described by Thomas and Harden [21]. Codes were inductively created during the coding process, as new ideas were identified, and previous ideas entered in relevant existing codes. After all line-by-line coding, codes related to each other were congregated into descriptive themes and subthemes, and then into analytical themes [21]. The aim of the analytical themes was to synthesize all the main ideas found in the selection of articles. After the articles were fully coded and themes identified, the coded data were verified and, if necessary, recoded to increase data representativity. To increase the analysis depth and validate the coding process, counterexamples of the identified themes were explicitly sought.

## Results

### Study selection and screening process

**Fig. 2:**
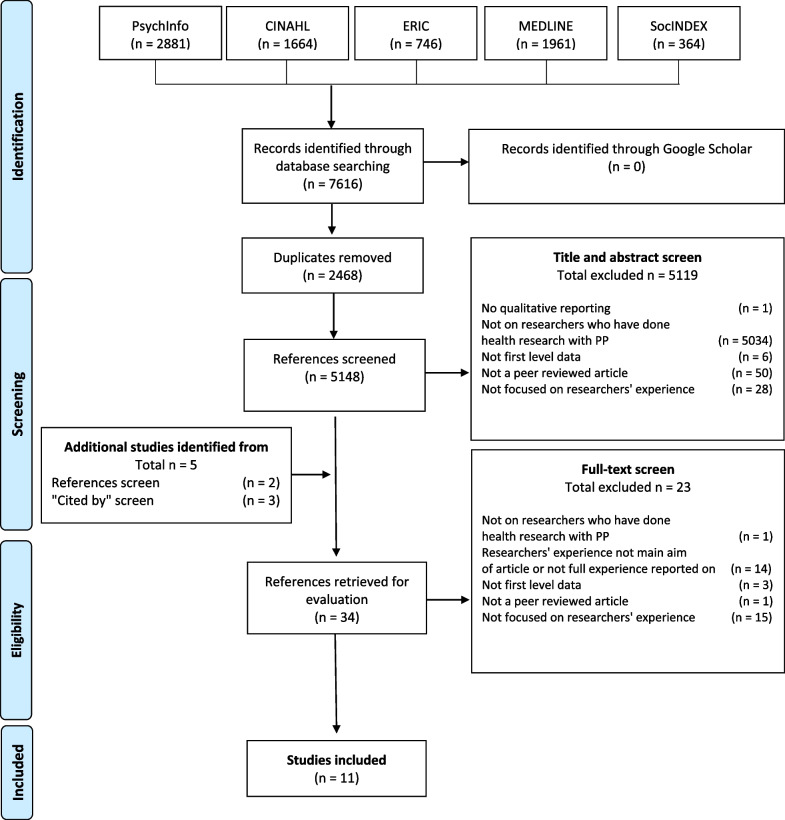
Flowchart of study selection and screening process

The search strategy yielded a total of 7616 results in selected databases, and no further results were found via Google Scholar. A total of 2468 duplicates were removed, either automatically by the referencing tool, or manually. A total of 5114 articles were found to be off-topic through the screening of titles and abstracts. The references and the “Cited by” list of the 29 remaining articles were screened, and 5 additional studies were identified. We excluded 23 articles after the full-text screening. The remaining 11 articles were included, as they met all the inclusion criteria (Fig. 2).

### Characteristics of the included studies

Most of the studies were conducted in the United Kingdom (n = 7) [11, 22–27] followed by Australia and New Zealand (n = 2) [28, 29], Canada (n = 1) [30] and Singapore (n = 1) [31]. They were published from 2009 to 2019, with a majority after 2014 (n = 9) [11, 24–31] which could be and indicator of the relative novelty of reporting on health researchers' experience with PE.

Most of the studies used interview verbatims as evidence sources (n = 10) [11, 22–26, 28–31], and one used excerpts from reports of funding use [27]. The studies included between 10 and 36 participants [11, 22–31]. Of the 11 studies, 4 reported the engagement of PPs in their study [25, 27–29].

In the selected articles, two pairs of articles present different analyses from the same series of interviews: Thompson et al. [22] explored the purpose, the reasons for and against, the experiences, and the impacts of PE in health research, whereas Ward et al. [23] studied the scope, limitations, key purpose, experiences, and arguments for and against PE while adding a critical perspective to their analysis. In 2018, Happell et al. [29] analyzed data through the lens of allyship, and in 2019 [28], they explored the reported experiences and divided them into barriers and strategies for overcoming them. A comparative summary of the main characteristics of the included studies can be found in Table 1.

**Table 1:** Comparative summary of the main characteristics of included studies

References, Country	Title	Aim of the study	Design and sample	Participants (n, demographics, work experience in PE health research)	Specific population (researchers)	Main findings
Allard et al. [30], Canada	What does patient engagement mean for Canadian National Transplant Research Program Researchers?	“describe CNTRP researchers’ perspectives on PE before and after their participation in a national priority-setting workshop with patients, caregivers and clinicians.”	“Qualitative exploratory design”, “Semi-structured interviews”, “Content and thematic analysis”	n = 10 Gender: 4 F, 6 M Research field: 4 kidney transplantation, 3 basic sciences, 1 multi-organ transplantation, 1 donation, 1 legal and ethical issues Province: 4 ON, 3 AB, 2 BC, 1 QC Work experience in PE health research: Before the workshop: 4 Yes, 6 No	CNTRP researchers	Patients’ experiential knowledge was viewed to enhance the relevance and quality of medical research within the CNTRP
Baxter et al. [27], England	Evaluating public involvement in research design and grant development: Using a qualitative document analysis method to analyze an award scheme for researchers	“to analyze reported views and experiences regarding the processes of public involvement, in order to investigate elements that might be barriers or facilitators to public involvement in research proposals”	Qualitative design Reports of funding use Analysis with group participatory approaches, qualitative content analysis and framework analysis	n = 25 (reports)	Health and social care researchers	Researchers recognize the variety of potential roles for public members in research and acknowledge how involvement adds value to studies
Boaz et al. [24], UK	Rethinking the relationship between science and society: Has there been a shift in attitudes to Patient and Public Involvement and Public Engagement in Science in the United Kingdom?	“explored researchers’ attitudes to both PPI [Patient and Public Involvement] and PES [Public Engagement in Science]”	Qualitative design Semi-structured interviews and card exercises about PE to stimulate discussion Inductive analysis, grouped in broad themes	n = 19 Level of experience: 4 professors, 1 reader, 1 senior lecturer, 2 research fellows, 3 research associates, 4 post-doctoral researchers, 3 Ph.D. students, 1 research assistant From three research area (one by center): genetics (6), mental health (7), health services research (6) Mix of basic biomedical scientists, health service researchers, clinician scientists (doing research and clinical practice) Work experience in PE health research: mandatory with working at Biomedical Research Centers	Researchers working in 3 Biomedical Research Centers	While researchers are usually open to the implication of PPs and its advantages, their role is understood as peripherical, as “science remains the preserve of scientists”
Boylan et al. [25], UK	“About sixty per cent I want to do it”: Health researchers’ attitudes to, and experiences of, patient and public involvement (PPI)—A qualitative interview study	To explore researchers’ experiences and perceptions of PE	Qualitative design Semi-structured interviews (theoretical domains framework) Iterative thematic analysis (theoretical domains framework + inductive code)	n = 36 Gender: 22 F, 13 M Ethnicity: 24 white British, 3 white European, 6 white other, 1 British Asian Age: 14 26–44y, 18 45–64y, 3 unspecified Role: 14 clinical and medical scientific researchers, 18 social scientists and health services researchers, 3 PE coordinators Working in: England, Scotland, Wales Variety sought for types of research conducted, research design, level of seniority, degree of experience doing and demographic (age, gender, ethnicity) Work experience in PE health research: 1 < 1y, 10 1–5y, 8 6–10y, 13 > 10y, 3 unspecified	Researchers	Participants expressed ambivalence, cynicism, and enthusiasm about the PE, an activity that is both rewarding and challenging
Happell et al. [29], Australia/NZ	“I don’t think we’ve quite got there yet”: The experience of allyship for mental health consumer researchers	“to explore the views and opinions of other mental health researchers about working collaboratively with consumer researchers, including strategies to further advancements in this area.”	“Qualitative exploratory design”, “Semi-structured interviews”, “Thematic analysis”	n = 11 6 F, 5 M Employer: 10 university, 1 non-government organization Country: 6 New Zealand, 5 Australia Positions: 3 professors (1 also director), 1 project manager, 4 senior lecturers, 2 associate professors (1 also director), 1 post-doctoral research fellow Disciplines: 2 psychiatry, 4 mental health nursing, 2 social work and 2 psychology, 1 discipline not mentioned Work experience in PE health research: purposive sampling of researchers known by the research team	Established mental health researchers	Health researchers find value in PE. They can support and facilitate engagement by encouraging active and meaningful participation
Happell et al. [28], Australia/NZ	“Chipping away”: non-consumer researcher perspectives on barriers to collaborating with consumers in mental health research	“to explore the opinions and experiences of other mental health researchers from Australia and New Zealand regarding working collaboratively with consumers in the conduct of research, with the view to enhance understanding of how consumer involvement in research could be increased.”	“Qualitative exploratory design”, “Semi-structured interviews”, “Thematic analysis”	n = 11 6 F, 5 M Employer: 10 university, 1 non-government organization Country: 6 New Zealand, 5 Australia Positions: 3 professors (1 also director), 1 project manager, 4 senior lecturers, 2 associate professors (1 also director), 1 post-doctoral research fellow Disciplines: 2 psychiatry, 4 mental health nursing, 2 social work and 2 psychology, 1 discipline not mentioned Work experience in PE health research: purposive sampling of researchers known by the research team	Mental health researchers	Barriers to PE include power disparities between researchers and PP. Developing simultaneous collaboration strategies for collaboration is the most effective and should be prioritized
Paul et al. [26], UK	Involving the public in mental health and learning disability research: Can we, should we, do we?	To explore attitudes of researchers working in mental health and learning disability services towards PE in research	“Qualitative exploratory design”, “Semi-structured interviews and reflexive journal”, “Framework analysis”	n = 8 Research role: 2 clinical studies officers, 1 research manager, 3 principal investigators, 2 clinical trials coordinators Research background: 6 randomized controlled trial, 2 observational study, 1 various, 1 feasibility study, 1 qualitative study Client group: 1 learning disability, 1 mental health, 6 mental health + learning disability Professional group: 2 medicine, 1 clinical psychology, 5 none	NHS (National Health Services) Foundation Trust staff involved in research	Even though UK has a history of PE practices and developed clear guidelines, challenges persist for full integration of PPs across the complete research process
Puerta et al. [31], Singapore	Researchers’ perspectives on public involvement in health research in Singapore: The argument for a community‐based approach	To explore how researchers working in health research understand the principles, role and scope of PE. Explore challenges and opportunities of PE implementation in Singapore	“Qualitative exploratory design”, “Semi-structured interviews and reflexive notes”, “Thematic framework analysis”	n = 20 Gender: 11 F, 9 M Ethnic group: 14 Chinese, 3 Caucasian, 2 Indian, 1 Malay Position: 1 research follow, 5 doctoral researchers, 2 GP researchers, 3 assistant professors, 3 associate professors, 2 research managers, 1 adjunct associate professor, 2 professors, 1 occupational therapist Research experience: 3 < 1y, 5 1-5y, 2 6-10y, 10 > 10y Research field: 2 pregnancy & parenting, 3 older adults, 2 neurology, 2 family medicine, 4 chronic conditions, 2 psychiatry, 2 general population, 2 physiology of ageing, 1 patients with cancer	Researchers	Singapore’ culture of participatory collective model has the potential to contribute and inspire more inclusive PE practices in research communities around the world
Staley et al. [11], UK	The impact of involvement on researchers: a learning experience	To explore “researchers’ experience of involvement and in particular what they learn from the process”	"Qualitative design", "Semistructured interviews", "Theoretical thematic analysis"	n = 8 Disciplines: 7 clinical research project, 1 basic science project Working in: England, Scotland, Wales	Researchers who worked in a pilot PE project by Parkinson’s UK	PE experiences lead researchers to acquire new skills and knowledge, as well as to change their values, their preferences, and their research practices
Thompson et al. [22], UK	Health researchers attitudes towards public involvement in health research	To explore “university health researchers’ attitudes and opinions towards public involvement in health research.”	“Qualitative exploratory constructivist design”, “Semi-structured interviews with constant comparative method”, “Broad interpretive iterative analysis loosely guided by grounded theory”	n = 15 Gender: 10 F, 5 M Research background: 10 health service research, 2 clinical trials, 3 biomedical/laboratory Academic grade: 1 research fellow, 1 senior research fellow, 1 clinical trials manager, 2 professor, 1 principal clinical lecturer, 2 research associate, 1 research officer, 2 research fellow, 2 lecturer, 1 senior lecturer, 1 reader Working in: England, Wales	University health researchers	Researchers’ understanding of PE varies. While the theoretical benefits of PE are understood, apprehensions, and uncomfortable feelings when implementing PE are common
Ward et al. [23], UK	Critical perspectives on ‘consumer involvement’ in health research: Epistemological dissonance and the know-do gap	To present “data from a qualitative study of researchers about their perceptions and experiences of consumer involvement in research.”	“Qualitative design”, “Semi-structured interviews”, “Interpretive analysis with open coding and categorization from grounded theory”	n = 15 Gender: 10 F, 5 M Research background: 2 health services research (qualitative), 2 clinicals trials (quantitative), 3 health services research (mixed methods), 2 health services research (quantitative), 3 laboratory research (quantitative), 1 primary care research (mixed methods), 1 population based research (mixed methods), 1 medical statistics (quantitative) Discipline: 2 social science, 2 medicine, 3 public health, 2 psychology, 3 biomedicine, 1 general practice, 1 nursing, 1 statistics Working in: England, Wales	University health researchers	A common issue of raises by researchers about PE is “the tensions between researchers’ perceptions of the potential benefits of, and their actual practices in relation to PP”

### Thematic synthesis

The analytical syntheses that emerged from the selected studies were classified into five central themes: (1) the understanding of PE, (2) the motivations for PE, (3) the contexts of PE, (4) the attitudes toward PE, and (5) the practical aspects of PE that are of central importance to researchers. We recognized that experiences cannot be separated into unique distinct categories, and that many statements lie at the intersections of those categories.

#### How is PE understood by health researchers?

**Fig. 3:**
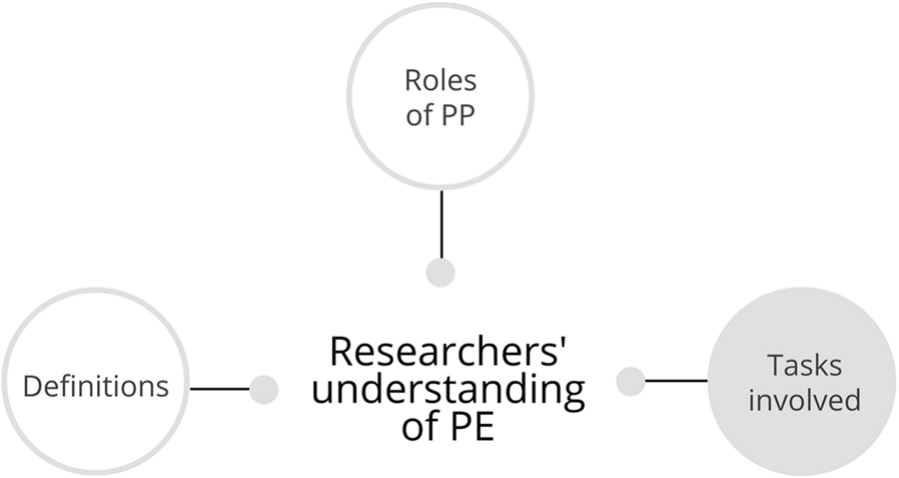
Researchers’ understanding of PE

A starting point that allowed comparison between participants’ experiences was how they defined what PE was and how they conceptualized the forms it could take (Fig. 3).

##### Defining PE

All authors—except Staley et al. [11], who did not offer a definition—defined PE similarly as “research conducted *with* or *by* members of the public rather than *to*, *about* or *for* them” [22–31]. Although most of the participants agreed with this definition of PE, some also expressed uncertainty about the purpose of PE [22, 26, 31]. Participants in one study perceived involving patients or the public as having participants in their clinical trial [22].

##### Roles of PP

Typical roles of PPs described by participants included advisors to the research team or part of an advisory group [22, 24, 26, 27, 30], giving feedback on research design and materials [11, 22, 27, 30], or co-researchers [24, 26, 30]. Roles that were described less frequently were overseers of the research conduct [27] or members of formal and stable research team in academic organizations [29].

Some participants stated that PE should occur at every step of a health research project [30]. More precisely, they could participate in developing research design [11, 22, 24, 26, 27, 30, 31], writing grants proposal [27, 30] or ethics applications [24, 27], validating the relevance of researchers’ work and acceptability of research [22, 23, 25–27, 30], engaging with patients [30], analyzing and interpreting data [27], writing findings [27], promoting public awareness of research [24], and disseminating and translating the research in practice [27, 30].

##### Tasks involved

For health researchers, engaging PPs in their research project came with administrative labor [24, 25] that was mainly described as the recruitment of PP, securing contracts, and obtaining ethical approval [24]. This administrative labor was referred to as a barrier to engagement, as the time to obtain necessary documents could exceed the time window for involving the PPs [24].

“I was doing a project where we were trying to get service users on board, and we had employed two, from the [local patient group], so we had gotten them on board, got honorary contracts for them through [the Trust], and then, of course, recruitment stopped. So, I was kind of left at a loose end of what to do with them.” [24]

Emotional labor was another task described in PE [25]. Participants described having to manage their feelings and emotional responses because of the presence of PPs. The illness of the PPs was a factor that could increase the emotional labor needed. Some participants explained that it was a rewarding experience for them despite the emotional cost [25].

“…emotion is the power that they bring to the situation.” [25]

“…It ceases to be an academic exercise, you’re working with real people…and something seemingly innocuous can just trigger something for somebody… You have responsibility as such to take care of the people you’re working with. And I think that’s a very personal emotional cost because these aren’t other researchers; these are patients and members of the public.” [25]

However, some health researchers were less enthusiastic about the emotional labor involved and felt less motivated to engage PPs in their research as a result [25].

“Mm, about sixty percent, I want to do it. The bits of me that don’t want to do it are the kind of, ‘Oh god I’ve got to be polite to people when I’m not in the mood to be’ [laughs] – all that kind of stuff.” [25]

#### What are PE motivations for health researchers?

**Fig. 4:**
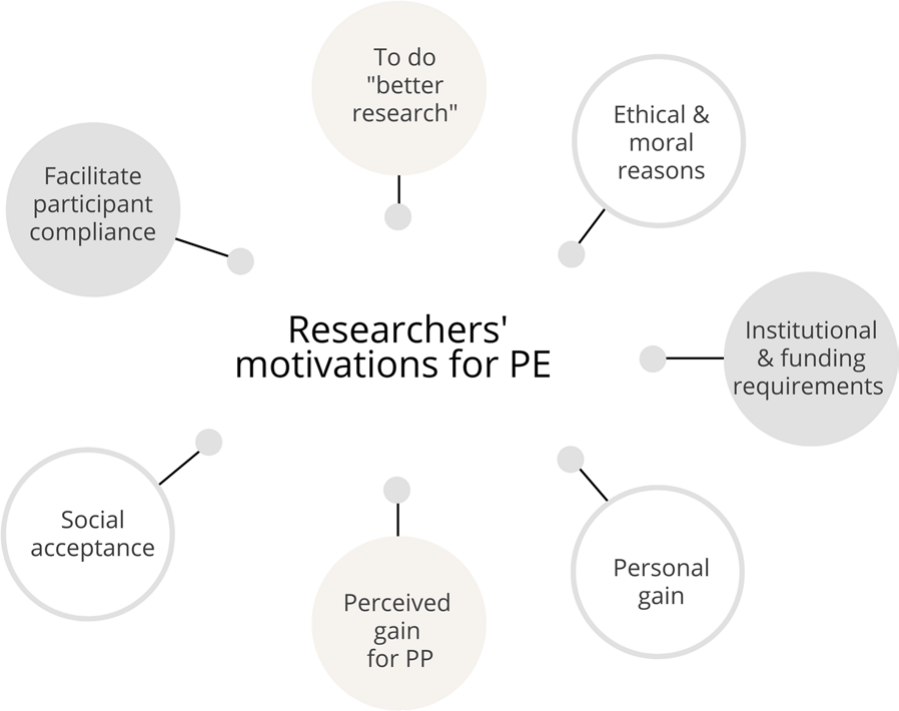
Researchers’ motivations toward PE

In discussing their experiences, participants mentioned different motivating factors: producing better research, ethical and moral principles, personal gain, perceived gain for PPs from the experience, requirements from institutions, improving the social acceptability of science, and having more compliant participants (Fig. 4).

##### To do “better research”

The participants shared how having PE in their health research led to improvements in many aspects of their projects. The improvements were mentioned as a general statement [23, 31] as well as precise descriptions of what PE brought to the researchers’ work [11, 22–27, 30, 31].

“If I don’t do this [PE], it might also lead to a lot more problems later … you know, you do it very quickly and then you come up with a design that is not good. Then, you actually have to do a lot of patchwork to try and solve the problems.” [31]

The presence of PPs was key to obtaining new perspectives [11, 27, 30] and new knowledge or perceptions [26, 30, 31] of their projects.

“They felt that research attempting to understand what people can do to maintain wellbeing would be very beneficial to patients.” [27]

“Comments were made that it [the research topic] resonated very strongly with their own experiences.” [27]

Participants reported that the engagement of PPs improved various aspects of their health research projects: research relevance [22–25], writing the research proposal [25, 27], setting research priorities [26, 30, 31], recruitment process [23–25, 27, 30, 31], increasing long-term participation [27, 31], research design (methods, intervention, collection tools, outcomes [11, 22, 24, 26, 27, 30, 31], ensuring research accessibility [26], ensuring accessibility of communication materials destined to patient participants [11, 25–27], raising potential ethical or patient safety issues [27] and dissemination of the search as well as translation in practice [23, 24, 27, 30, 31].

Health researchers perceived that PE led to better research quality in many ways, such as improving research proposals [27], improving the accessibility of communication materials [11, 22, 25–27], improving research priorities [26, 30, 31], improving recruitment [23–25, 27, 30, 31], leading to more long-term participation [27, 31], improving research design [11, 22–27, 31], and creating more empirically useful research [11, 23–25, 31].

##### For ethical and moral reasons

Participants also evoked ethical and moral principles as a motivation for choosing to engage PPs in health research [22–27, 30, 31]. A major argument in this category was the patients’ right to have their voices heard [11, 22, 26, 30], even more in public-funded health research [26].

“…if we’re doing publicly funded research, then the public have a right to steer that, I think.” [26]

“I’m not sure how to sum it up in one sentence, but I think the key purpose is to take the viewpoint of the people you are researching, and to not use them as subjects but as equal partners in the research, as far as you can, because I think there’s far too many times when research is done to people and they haven’t been able to inform it, and their views should be taken on board and are very valid. So essentially, I think it’s about power relations in research; it’s about respecting the people that you’re researching because I don’t think you can just come at it from one angle when you’re not in the shoes of the people that you’re researching.” [22]

Similarly, they explained that the context of resource scarcity [30, 31] further highlights the need to focus health research efforts on what is relevant to patients and the public [30].

“We should be using [the] scarce research dollars to study and understand questions that are relevant to our patients.” [30]

“And [PE would] probably save a lot of money for the grant bodies.” [31]

The need to represent different individual experiences of health care [27] was also mentioned, as was the necessity to ensure the accountability of studies conducted [23–25, 27]. The argument that the presence of PPs humanizes health research was also made [25, 30].

“[It’s] my work – to someone else it’s their everyday, it’s really emotional, a big issue for them.” [25]

##### To fulfil institutional obligations and/or funding requirements

The requirement of PE by funding guidelines was cited by participants as a motivation to engage PPs in their health research [22–24, 26, 30, 31]. This requirement was experienced by some health researchers as another hurdle in their funding process [24], increasing the risk of their participation lacking meaningful engagement [22–24, 30].

“You have to be careful not to use patients […], that’s the risk. I think you have to be extremely vigilant about that, because it’s a fine line, I think, between honestly involving patients, having them truly participating […]. I believe you have to really be vigilant and attentive.” [30]

However, other participants highlighted that these requirements could also be a positive motivation to include PPs in their health research [22, 26, 31].

“…so those nudges and those requirements…make sure you have thought about it.” [26]

Others mentioned that they viewed this requirement as advantageous for receiving funding for the type of research they are already working on [31].

“Because if we are talking about translational research and that’s your area of study, showing that your study is centered around your patient is the number one criterion.” [31]

##### For personal gain

Although not described as a motivation for initially engaging with PPs, the knowledge and skills gained by the health researchers during these partnerships seemed to motivate them to maintain the relationship. Participants highlighted that the PPs helped remind them of what was important in their work [30] and that the partnership gave their work meaning [11, 25, 30].

“There is a personal aspect that must not be understated. You realize that the work that you’re doing is very important to people… sometimes when you work, things don’t go your way, there’s problems. It gets really frustrating, and you think, ‘What’s the point?’ The pay isn’t great, the funding is hard to come by, the job security is sometimes difficult… but then I realize… there is actually quite a lot of importance behind the stuff that I do for people – that is of value to me – it makes me feel good about what I’m doing.” [11]

The presence of PPs enriched the research process by making it more fun, giving the health researchers enthusiasm, or leaving them energized [25].

“…It raises my enthusiasm to battle the challenges of getting research funding.” [25]

Lastly, the health researchers mentioned that having better research helped with their career development [25].

##### For the perceived gain for PPs

The researchers also spoke of PE in health research as rewarding for PPs. This also motivated researchers to continue involving PP. It was perceived as empowering for the patients through acknowledgements of their experiences and opinions [28, 30], or the contribution of new knowledge to health research [26, 30]. Paul et al. [26], who focused on mental health and learning disability researchers, also reported that participants thought it could help PPs in their personal recovery by providing a new perspective on their experiences [26].

“It can almost be a beneficial part of recovery as well because it’s turning what was probably something quite negative about their lives into something quite positive because they feel like actually this is something that’s valuable about me, you know, this experience is worthwhile sharing and does mean that I can bring something. It’s not a part of their life they have to write off.” [26]

##### To enhance the social acceptability of science and health research

Participants evoked an interest in promoting a better understanding of health research and medicine to the general public, and PPs were recognized as advocates who could get engaged in ways researchers could not [22, 24, 30, 31].

“the lay public also becomes [a] tremendous [advocate] both in the media and among the larger community for research, because they become invested in it in a way that the medical health professionals and professional researchers [cannot, being] a little bit more divorced from directly accessing that community other than as guests in the community.” [30]

##### To facilitate participants’ compliance

A motivator shared by one health researcher in the sister articles by Thompson et al. [22] and Ward et al. [23] was that PE could make participants more compliant if they knew and understood more about the research process [22, 23].

#### What is the context of PE for health researchers?

**Fig. 5:**
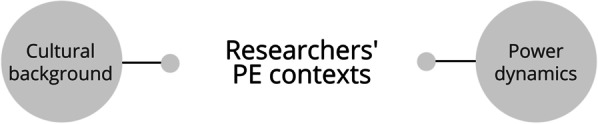
Researchers’ PE contexts

Although we focused on health researchers with an academic background, having a variety of studies meant that the participants were engaging PPs in their own different contexts, as defined by their own culture and power dynamics (Fig. 5).

##### Cultural background

Health researchers need to navigate the culture in which they work. These cultures can be either barriers [22–26, 28–31] or facilitators [25, 26, 29] to PE. Different societal cultural norms will also impact the dynamic between the patient and health researcher. For the participants based in Singapore, PE was seen as a barrier compared to the perspectives of health researchers in countries outside Asia [31].

“It’s just cultural. I think it’s like that student‐teacher, doctor‐patient type hierarchy relationship. So, a student will never argue with their teacher. So, the teacher can never be wrong. In the same way, the doctor can never be wrong. Whereas, I think in jurisdictions maybe outside Asia…it’s quite common for patients to verbalize what they feel […] Because of the culture [in Singapore], patients just don’t speak up about what they need… [they] rarely question doctors.” [31]

Cultural barriers can also emerge from the organizations employing health researchers’ [22–26, 28–30] as a lack of support for PE from senior colleagues [22].

“… Don’t underestimate how tricky it can be when a more senior person has … more set views on what PPI can and can’t do … If someone in a more senior position isn’t willing to open their mind and be receptive to genuine change, isn’t willing to accept differences to what they want to do, then there’s kind of no point in you trying really because I think everyone needs to be working to the same goal for it be effective.” [25]

The value that organizations place on PE was sometimes perceived by participants through many lenses. PE was seen as a requirement that could be satisfied by minimal effort from the research teams [23–26].

“There’s no pressure to do an amazing job on it because people aren’t expecting a lot from it…” [25]

Participants also mentioned organizational cultural barriers that made health researchers question the value organizations placed on PE, including the accepted and unchanging overwhelming workload to which PE is added [22, 25, 26, 30], the publish or perish culture [25], or the lack of resources available to them [25].

“I don’t get any extra allowance to do PPI… it’s just something extra that I’m having to fit on top of everything else.” [25]

The health researchers’ roles and responsibilities are shaped by each organization’s culture. The nature of contractual employment in health research teams was seen in tension with the task of establishing a partnership with PP [25].

“…if you’re on a fixed‐term contract of twelve months or six months, it can be quite hard to build a relationship that’s meaningful.” [25]

The health research community culture and identity also had an impact on PE because the specialized language, which is a common practice, was also outlined as a barrier that can exclude PPs [26, 30]. The participants expressed that no consensus was reached about PE among the health research community, and the divide was more important depending on the type of research conducted [22, 24–26, 30].

“[P]atient engagement isn’t going to apply in every setting and situation. […] I could see how a basic scientist is going to see very little use for this. So again, it depends on the perspective and the type of research.” [30]

However, many participants saw no limit to PE [24, 26, 29, 30].

“I see very few studies where it wouldn’t be helpful.” [30]

Cultural aspects could also facilitate health researchers’ activities with PPs. Colleagues’ buy-in and experiences with PE could also offer unique support that helped the health researcher’s experience [25, 26, 29, 30].

“…There’s a cultural support for involving various patients and members of the public in the organization, and I think that’s very helpful to be part of. And even kettle conversations whilst making a cup of tea and you’re sharing challenges and experiences with people who understand that, are a great resource, so I do think the culture of the organization is an important support for being able to do this properly… It would be much more difficult if you were on your own doing this without a supportive community of people.” [25]

Organizations’ and funding bodies’ support of PE also played a role in encouraging health researchers’ engagement with PPs [25, 26, 29].

##### Power dynamics

Power dynamics on different levels were a recurring theme described by participants [25, 26, 28, 30, 31]. They occurred between the health researchers and the PPs [26, 28, 30, 31] or between the research team members [25, 28]. The different values and importance attributed to academic knowledge and experiential knowledge were reported as barriers to implementing or valuing PPs’ contributions [26, 28, 30].

“I think that some research teams are very hierarchical, quite outside of anything to do with health … just have a very hierarchical process, and so they are more likely just to notice the seniority of who’s in the room. And often the consumer researchers are pretty junior, so they’ll get pushed to the research assistant status, or the junior researcher position.” [28]

Participants also talked about this power dynamic from the PPs’ point of view, reflecting that they could feel inferior to health researchers and clinicians [30].

“I think, on the one hand, […] the academic people—so the clinicians and the scientists by virtue of […] what we do and who we are—there might have been a bit of a power dynamic in the room. And […] for myself, I would certainly feel very free to speak, whereas […] the patient participant might feel more intimidated to speak.” [30]

Some health researchers still saw patients as vulnerable [30], while others were critical of how colleagues would consider them passive subjects [31].

“[T]here is obviously a power difference, power distribution issues, especially if you involve patients; they are vulnerable and, of course, physicians have the relevant power in the health care, so that’s somewhat unequal, so that has to be somehow balanced out. [sic]” [30]

“In taking part in research, they feel very much like experiments or they feel like a laboratory rat or a guinea pig… that disempowers them.” [31]

Among team members, the power dynamics could be influenced by the hierarchical status between professional disciplines [22, 25] or seniority [25, 28]. The social and interpersonal skills needed for successful PE were described as soft skills by participants and could lead to unequal distribution of added responsibilities. These could be given to women or newer team members. Participants reflected on men being supportive of PE in health research but not doing the actual work needed for the engagement of PPs [25].

“I find it interesting that so much of the researchers who are involved are female. You go to [academic] meetings about public involvement and you get one man and 20 women, and is that because…. they’re [sic] softer skills about communication and listening and empathy?” [25]

Although PPs were often described as simply subjects to the power dynamics in play, PE and the empowerment it brought to PPs were seen as a counterpart [22]. These power dynamics and cultural contexts that health researchers need to navigate while learning to include PP in their research can influence their attitudes toward PE.

#### What are health researchers’ attitudes toward PE?

**Fig. 6:**
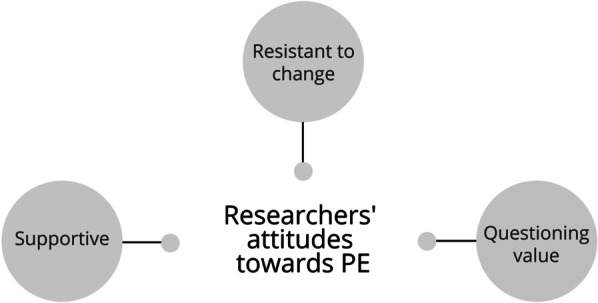
Researchers’ attitudes toward PE

Health researchers have different reactions to PE. Whereas some are very supportive, others question the value of such collaborations, and some are resistant to changing their usual way of thinking and doing health research (Fig. 6).

##### Supportive of PE

Health researchers expressed general support for the concept of PE and its benefits [24] while reflecting on their own personal experiences [11, 24, 30]. Researchers who were supportive of PE also believed that sharing control with PPs was possible [24] and that PE could bring necessary changes to health research [28]. Support for PE was also put into action by a health researcher who helped a PP to secure a long-term research position so they could continue their work [29].

“I was very impressed with the work that she had done on her PhD…And it seemed that it would be a great shame if this work came to an end, and I managed to find a bit of money to support her having a role [research and teaching] and eventually I managed to persuade our dean [for a] permanent appointment rather than a year by year arrangement…There was a bit of resistance to some of her initial fairly forthright views about what we weren’t doing right…I think that was fairly important that I’d been around the department a long time and … it was quite clear that I was supporting the line she was taking if not necessarily every element of it. And people were willing to take a bit of a lead from that and not want to be seen as rejecting.” [29]

Another concrete way a health researcher supported PE was by taking the responsibility of providing funding through consultancy work to inject into PE projects [29].

“…what I try to do is squirrel as much resource together as I possibly can … sometimes a little bit of consultancy work which gives me some money, or I do projects, which I know at the end of the day … have a little bit of money surplus at the end … I can have a little bit of control over that resource, so then I take it back to the [consumer] groups that I’m negotiating, that I’m working with and say to them, “Okay. What is it that you want us to have a look at, and here’s a pot of money that can assist it?” It might not get us completely there, but it can start the process.” [29]

Acts of support were also obvious among health researchers who shared their knowledge of the new organizations in which PPs ended up, helped organize PP networks [29], and advocated for PE [28, 29].

“So the notion of co-production in service user led research is essentially in its infancy … And so what you need are some people who just stand out there and say not only this is important, but this is the absolutely most important thing ever …it’s a challenge of doing any work that’s in its infancy, as that you may need to take a more radical position than you may otherwise, simply to garner traction …the problem with caution with research in any area that appears to be in its infancy is it just goes nowhere.” [28]

One of the attitudes of PE-supporting health researchers reported in many studies was the expressed acknowledgment of the added value PPs bring to projects. They recognized PPs as having valuable knowledge to share, an expertise considered complementary to health researchers’ knowledge [11, 24, 27, 30, 31].

“Because researchers themselves, you don’t know everything. You don’t know what’s on the ground level; the daily operations, challenges and things like that.” [31]

“In my view, it is a mistake that we just look at the evidence…sometimes, as researchers… you no longer hear right what people are saying… We think we know because we read what our peers publish in the journals, but do we really know?” [31]

This value was also acknowledged by actions from health researchers, such as providing them with feedback on how their contributions influenced the researchers’ work [27] or involving them even more [24].

“[We] have become very aware of the need to involve users and participants, and we’ve sought their advice and help to design better studies, to help with the recruitment, to help understand the experience of the subject of research, so we’ve been involving them all the way along.” [24]

A way health researchers’ supportive attitudes were developed was through having a positive experience of PE. Health researchers who had a positive experience with various PP activities expressed more support for PE than before [11, 24, 30]. This was clearly exemplified in Allard et al. [30] interviews with health researchers before and after a workshop involving PP.

“… being [a] basic scientist, I’m very far away from patients, so usually I don’t see patients, I don’t interact with them. It was really nice to hear their stories and to hear what they thought was important. And I think it definitely opened my eyes as well from a basic science point of view and in […] how can we further engage patients in what we’re doing and [have] their opinions, but also […] inform them better about what we’re doing and how much time things cost, and, you know, that it’s a better understanding from both sides.” [30]

Such positive experiences allowed them to gain first-hand experience of the value PPs bring to health research and how it was possible to put it in place [11, 30].

“It was a bit of an eye-opener for me, and it brought me more in line with what [patients] would deal with on a daily basis… It was really small things that I would have taken for granted… things that you would almost ignore, really, in the design of the trial.” [11]

Some participants had their views and assumptions challenged by PPs, which were positively experienced as well as perceived as a powerful experience [11].

##### Questioning PE value

Although many of the participants were supportive of PE, some researchers were not convinced of the value of engaging PPs in their health research [22, 23, 26, 30]. They questioned the amount of work that needs to be accomplished to engage PPs in contrast to its perceived benefits, citing a lack of data [30].

“I think it’s a lot of work to bring patients up to speed on the process and the content; it takes a lot of work. And it’s unclear whether the benefits of putting that much time and effort in are going to add value.” [30]

The possible ethical or impractical implications of PE lead health researchers to have hesitations. However, the following participant did not see this as a reason not to engage PPs, but to be cautious in setting it up [26]:

“…there are a lot of questions that the public and patients would like to ask and it’s very difficult to do that methodologically or operationally… on the one hand you want to encourage people to take up the opportunity of talking and developing their idea… but then, you know, there are things that are actually very impractical, may be unethical… so we don’t want to give people unrealistic expectations, I suppose.” [26]

A participant who is a clinician evoked having daily contact with patients and therefore didn’t feel the need to engage PPs in their health research [22].

“Well, I mean I’m a GP, so I am sitting and listening to what patients are telling me every day. So it is less relevant, I think to me. I think it is much more relevant to the non-clinical researchers actually. And especially the non-qualitative, non-clinical researchers, you know, some of our epidemiologists and perhaps statisticians and health care service people.” [22]

Other participants did not negate PPs’ experiential knowledge but felt that *“their experiences cannot outweigh my academic qualifications or knowledge”* [23]. Some participants reported discomfort in involving PPs because they could bring their own agendas and be a threat to the health researchers’ work [23, 30].

“I mean, of course, they’re coming to the table because you want to know, you want the benefit of their experience, but if people have an agenda going into those conversations […] it can become all about their agenda rather than about the actual research program that is being developed, right? So that’s definitely something that needs to be avoided, because that can be very destructive and frankly time-consuming, again, when it comes to the scientist.” [30]

Researchers who questioned the value of PE also perceived engaging PPs in health research as inappropriate. Sometimes, this emerged from perceiving PPs as a vulnerable party that needs to be protected [28, 30], not having the capacity to participate significantly in health research [23, 24, 26, 30], or not being representative of patient and public realities [23].

“Absolutely not necessary… It’s your paper, not their paper… They are subjects.” [31]

The possibility of PPs not being representative was expressed by some participants as a concern about presenting PE as a homogeneous voice, while some groups could be excluded. Some health researchers questioned the validity of PPs while raising the issue that when PPs acquired research experience and sometimes professionalized themselves, it made them less able to be a representative of PP experiential knowledge [23]. Ethics committees could also perceive PPs as vulnerable, and health researchers reported an instance in which they had to advocate against what they viewed as a paternalistic view of PPs [28].

“[…] the Committee needed to be convinced to look past their narrow view of mental health consumers in terms of vulnerability and symptoms: … we had quite a battle with ethics. I think we’ve got a super conservative ethics committee… they couldn’t critique the idea of people with lived experience having expertise to do the role, but came at it in another way… So it was clear they were uncomfortable with lived experience researchers, yet it was a very tight presentation that was referenced and theorized, and all the rest of it, so then they … picked up other things which were totally ridiculous, like you cannot give these participants who have got drug and alcohol problems and other things vouchers that they can buy alcohol with – it’s like, yeah, okay.” [28]

Some researchers justified that PE significantly depended on which type of health research was done [24].

“I guess, I’m also a bit scared of this idea of handing over some of the power and control to the public so they can influence how research is conducted, because, I feel like the decisions would be quite naive. It may not necessarily be in the best interests of research progress or, you know, getting a new drug or something like that.” [24]

The value of PE activities was questioned by health researchers who sometimes felt diverted from their usual activities and could put them at a disadvantage compared to their colleagues [25]. Having to engage with PPs was perceived by some participants as another obstacle to research approval [22].

“I definitely would want PPI to be involved in all my studies, but I don’t want to be the sole person responsible for every time because that’s going to take away from research time and then I’ll be doing the PPI for my colleagues and they’ll be able to do more research and get more publications out of it…” [25]

##### Resistance to change

Some health researchers expressed resistance to the changes PE brings to their work [22, 24–26, 28, 29]. One of the reactions was resistance to the idea of sharing control and power [22, 24], but some were comfortable if this was done for only parts of health research [24].

“So I think for me, it’s absolutely fine for patients to have enormous power over the direction of research and what the questions are, but the technical sides of it are I don’t think appropriate for patients. And then you get confused about the sorts of patients you’re attracting, and what actually is a lay person if they’re someone that’s capable of carrying out a scientific study. …So I think it’s just a mixing up of roles that isn’t terribly helpful to anyone.” [24]

Some participants expressed being uncomfortable or having apprehension about the process of PE because they felt it would lessen the value of health researchers and their knowledge [22, 24, 25].

“I’ve got to be really careful as to what I say and do … PPI’s really trendy at the minute … Patients should be researchers – why don’t we just [effing] bring a load of patients to come and sit round my desk? Why did I bother doing a PhD? Do you know what I mean? So it’s like really difficult because these people are quite capable people, but they’ve not had the training; they’ve not worked as a researcher.” [25]

Some participants experienced the requirements of involving PPs as a professional and personal insult [25].

“[There is] this idea that we need PPI because actually we’re all these kind of robotic, unfeeling people who don’t understand what patients go through …I’ve spoken to hundreds of patients; I spend all my time… exploring the impact on them, and you’re telling me that I don’t know anything about it… It’s almost a bit of a professional insult and a bit of a personal insult.” [25]

Other participants reflected that these types of reactions could come from insecurities [28], and were open to receiving criticism [29].

“…if you’ve got an insecure group, they’ll say, “Well, what are these service users doing taking over. This is our job.” [28]

“…probably like a lot of people that are working in his space …we all believe in what we’re doing, we all probably come from a mutual set of values around supporting each other and working collaboratively… and valuing good relationships …I don’t think I’ve necessarily always got it right …what is missing from this space is really the negotiations where we, as researchers, are firmly embracing what …you [consumers] tell us needs to be researched. I don’t think we’ve quite got there yet.” [29]

Certain participants were more critical of how the health scientific community conceived of their profession [24].

“I don’t think there is a lot of humility in the scientific community about their own need to be exposed, to…, because there is a certain elitism that floats around these circles in which people think they know the truth… So it’s not that they’re dying to get input from others and widen their perspectives.” [24]

“I think there is very much a normative ideal that actually professionals are the only ones that really have the authority and knowledge to write research protocols and undertake it.” [24]

One participant felt that giving up their control was a positive experience [26].

“It’s, you lose control of some of it, really. I think it is probably quite an exciting, provoking process, and it is hard work if you’re going to do it meaningfully.” [26]

#### What practical aspects of PE are important to health researchers?

**Fig. 7:**
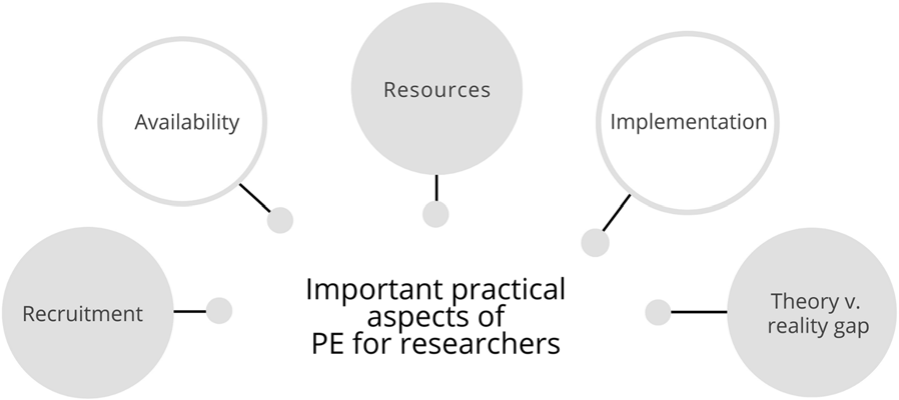
Important practical aspects of PE for researchers

In discussing PE, health researchers often mentioned practical aspects that were important to them and were decisive in their comprehension of how they could or could not integrate PPs. Aspects such as PP recruitment and availability, (un)available resources, implementation capacities, and any gap perception between theory and reality are examples of recurrent researchers’ discourses (Fig. 7).

##### PP recruitment

Health researchers shared their experience with PP recruitment concerning the techniques they used and the challenges they faced. A highlighted challenge of the recruitment process was ensuring suitable representation [23, 26, 30], but some participants accepted potential inadequate representation, as these PPs were “*more representative than nobody*” [23]. Health researchers also reported recruitment difficulties [24, 27], PPs dropping out during the process [27], and concerns about over-burdening the limited number of PPs involved [23].

##### PP availability

Difficulties with PP availability, since their engagement in the research project might not be their priority, were mentioned. Being unavailable can come from illness or other engagements, such as their full-time jobs [23, 27, 30]. This was experienced as a barrier to PE.

##### Resources needed

Participants identified a lack of resources to successfully engage PPs in their health research as an important barrier. They pointed to a lack of administrative support [25], time within their research timelines [23, 25, 26, 31], additional dedicated funding [23, 25, 26, 31], infrastructures [23, 25–27, 29, 30], and knowledge and skill competencies of health researchers and PPs [25, 27, 30, 31].

Some participants felt that they lacked the skills and knowledge to meaningfully engage PPs in health research [25, 27, 30, 31]. Boylan et al. [25] identified numerous skills relating, for example, to relationships, communication, and organization as necessary for a positive PE experience. Not knowing exactly what PE is was cited as a lack of knowledge on the health researchers’ part [30, 31], as well as an indicator of missing guidelines and infrastructures in the responsible organizations [30].

Missing infrastructures were described by many participants [23, 25–27, 29, 30]. Some health researchers called for more infrastructure in a general manner surrounding PE [22, 31] while others described what they desired in more detail. A need for clearer expectations of PE from organizations in the form of guidelines, guidance, or a framework for health researchers [25, 26, 30], as well as grant reviewers [30].

“I think that it needs to be […] focused, we need to have an idea of what specifically we’re hoping to achieve through PE and have […] a plan as to what stage in the research process it might be appropriate to bring patients in, and really have a sense of what we’re hoping to achieve with that level of engagement.” [30]

Participants recognized a need to support PP engagement and compensate for their time and travel expenses [27, 30].

“To facilitate time, I think these people need to be paid for their time. […] But figuring out a way of, first of all, a standard amount […] that everyone gets paid and some way of doing this easily… Because most of us don’t have extra research dollars for this. To figure [out] a way to facilitate their hours, travel for face to face.” [30]

Participants also made calls for organizational PP support that could take the form of scholarships and fellowships for PPs [23], or committees formed by PPs that would support PP [30].

Participants expressed that training for health researchers was necessary [23, 25, 30]. Having dedicated team members, such as PE coordinators, facilitating the engagement of PPs could be key to PE success [25].

“There’s a particular skill in being able to span those two worlds, the academic research world and the lay world and to act as some kind of translator between the two and I think there is something there that’s a particular skill…” [25]

Many participants felt that the barriers they experienced in engaging PPs could be addressed by providing them with training prior to or during research projects [24, 27, 30]. They saw that training sessions could help inform PPs about research processes, research methods, and scientific jargon, which would, in turn, empower PPs and make them feel more confident when they participate [27, 30].

##### Strategies for implementing PE

In describing their experience, participants suggested different strategies they used to facilitate PE implementation: fluid membership [27], local recruitment strategies [27], starting PE activities with the public before research proposal submission [27, 31], building trust [31] and keeping them informed during the project [22], considering prior training and providing financial compensation [27], communicating reminders via text messages instead of only using email as a form of communication [27], and adapting their language [26]. They also identified two helpful skills to develop: facilitation skills [27] and strategic leadership [31].

“Yeah, not using jargon all the time and fancy scientific terms when there’s plain English to do the same job.” [26]

##### Theory versus reality

Regarding PE implementation, several participants expressed a distinction between the theory and the reality of PE [22, 23, 25, 26, 28, 29]. Some participants suggested that the process of implementing PE should be done according to the level of participation available in their organization and then built to reach a higher level of participation [29]. They warned about the risk of tokenism at both ends of the participation level [29] and highlighted the gap between their aspirations regarding the policies for PE and their practice [23, 26, 29]. This gap was sometimes explained by the lack of resources they faced [26, 29] or the difference between what was mapped in the grant proposal and the real project [22].

“I don’t think we’re there [involving consumers fully in research], I think we are a million miles away from getting there at the moment, but I think that’s a nice utopia to aim for. But yes, I’m convinced by the hypothetical arguments. I just think we’re a long way from having any sort of infrastructure in place that would allow that to happen very easily.” [23]

## Discussion

The objective of this scoping review was to analyze the existing qualitative literature on the experiences of researchers in health research who have involved PPs in their research projects. The 5 main themes, which were inductively extracted from the 11 studies, provide an original synthesis of the experiences of researchers involving PPs in health research.

This scoping review has indeed brought light on how many health researchers have positive experiences with PPs [11, 22–31] and find that they bring unique value [11, 24, 27, 30, 31]. That finding is in line with extensive reporting about the multiples benefits of PE in health research [1–10, 32]. Researchers first good experiences with PE also bring more enthusiasm toward PE in general [11, 24, 30] which underlines the importance of organizations creating and supporting those first opportunities. However, the selected articles have also shown that some health researchers’ resistance to involving PPs is linked to the interpretation that PE would reduce their professional skills [25]. Part of this may be explained by a common dynamic of change resistance [22, 24–26, 28, 29].

Researchers are embedded in the health research culture in which they work. These are often structured without any consideration for PPs in the daily business of what ‘doing research’ entails [22, 25, 26, 30]. Integrating PPs asks for a change of perspective, and in some contexts brings a whole paradigmatic shift, as this is not how “sound research” has been understood, shared, and conceptualized in the past [22, 25]. From such a perspective, the mere idea of giving importance and carving space for the “mundane” in the “sacred” ivory tower of health research is easily perceived as counterintuitive, resulting in resistance. The top-down premise of “doing research to help patients” takes a toll when shifting the perspective to co-creating health research in a horizontal fashion *with* those directly concerned, not *for* them as experts in the field. This is an important change in self-identity and role definition. In some health research contexts, we can hypothesize that resistance to PE may be an unconscious effort by individual researchers to maintain cognitive balance. Further, given the common high workloads, high peer competition, and lack of resources (time, financial, and human) in research contexts [23, 25, 26, 31], it is unsurprising that taking the time to fully understand PEs, change one’s inner perspectives, rethink one’s professional identity, and adopt new practices may cause some degree of resistance. The testimonials of participants in this scoping review highlight organizational changes needed to support researchers working with PPs to allow for the sometimes invisible labor needed to address power dynamics, to create a supporting workplace and to engage in the emotional labor of redefining their professional identity.

There is a tension between the idea that PPs’ “experiential knowledge” may be diminished by any research training or “professionalization,” as if their life experiences could not be pure, authentic, or valuable if they decide to increase their knowledge about health research [23]. However, training PPs is often seen as positive in reducing power imbalances and barriers, such as scientific jargon and misunderstanding of research processes [24, 27, 30]. The idea that increased research knowledge could lead to reducing the worth of past experiences or one’s capacity to bring forth such experiences does not seem to hold ground.

## Strengths and limitations

The strengths of this scoping review include the rich data on researchers’ experiences with PE in health research settings, as well as the rigorous search strategy, extraction, and analysis of selected articles. This led to a rich summary of diverse experiences and perceptions on the topic, therefore providing a useful review for all interested in PE.

This scoping review also has a few limitations. The terminology inherent to PE is not standardized; thus, an exhaustive search on the topic can be challenging. To mitigate this, we worked closely with an academic health librarian at Université de Sherbrooke to develop an extensive search strategy, and many tests were conducted before the official and final extraction of data. We also narrowed our review to peer-reviewed articles, which are common for scoping reviews. We believe that further complementary research on a broader or differently limited scope (for example, on peer researchers, community researchers, or consumer researchers) would be a great addition to the analyzed data [15, 21, 33] and would serve the advancement of understanding PE.

## Conclusion

This scoping review provides a better understanding of the in-depth experiences of researchers in health-related research who have engaged PPs in their research projects. Through the thematic synthesis of many different aspects of these experiences, this review sheds light on various discourses relating to these experiences. The common advantages and difficulties relating to the unique perspectives of health researchers on this topic provide valuable information for a better understanding of how PE truly unfolds in different settings. Such insights could inform how PE can be supported and bring more positive experiences for all involved. The common hurdles to PE must be overcome if we are to adopt a conscious paradigmatic shift in research cultures to perceive non-researchers as having knowledge that is as invaluable as that of renowned experts in the field, capturing both expertise as complementary for leading the best health research possible.

## Supplementary Information

**Additional file 1:** ENTREQ statement checklist.

## Data Availability

All data and materials used for this study are available from the corresponding author upon reasonable request.

## Abbreviations

UK: United Kingdom
PCORI: Patient‐Centered Outcomes Research Institute
PE: Patient engagement
PP: Patient-partner
PPI: Patient and Public Engagement
ENTREQ: Enhancing transparency in reporting the synthesis of qualitative research
EQUATORnetwork: Enhancing the Quality and transparency Of health Research
APA: American Psychological Association
CINAHL: Cumulative Index to Nursing and Allied Health Literature
ERIC: Education Resources Information Center
MEDLINE: Medical Literature Analysis and Retrieval System Online
